# How an economic and financial perspective could guide transformational adaptation to sea level rise

**DOI:** 10.1038/s44168-025-00297-4

**Published:** 2025-09-24

**Authors:** Kees C. H. van Ginkel, Bart Rijken, Marco Hoogvliet, Wesley van Veggel, W. J. Wouter Botzen, Tatiana Filatova

**Affiliations:** 1 Deltares, Delft, The Netherlands; 2 Institute for Environmental Studies (IVM), Vrije Universiteit, Amsterdam, The Netherlands; 3 TU Delft, Delft, The Netherlands

**Keywords:** Climate-change impacts, Economics, Engineering

## Abstract

Accelerating sea level rise puts pressure on coastlines globally. Wealthy urbanized deltas like The Netherlands have historically followed the strategy of building increasingly large flood defence systems. But there is growing attention for ‘transformational’ alternatives such as adapting (‘accommodating’) land use to increasing flood risk. Until now, debates on coastal adaptation strategies have focused on aspects in the realm of the natural and engineering sciences. There has been limited attention to broader financial and economic arguments such as the increased financial risks associated with ever higher flood defence systems or the potential real estate devaluation of an ‘accommodate-retreat’ strategy. We advocate for a dialogue in which such economic and financial mechanisms are acknowledged, understood and thoroughly integrated into adaptation discourse.

## Introduction

Worldwide, the pressure from climate-induced sea level rise (SLR) on urbanized coastlines is growing. The ‘very likely’ range SLR scenarios (<3 m in 2200, 90% confidence interval)^1^ constitute significant biophysical and socioeconomic adaptation challenges. Meanwhile, high-end SLR scenarios (3–8 m in 2200)^1^ from icecap instabilities, pose even greater threats. Historically, wealthy urbanized coasts have typically adapted by building increasingly-large flood defense systems^2^. This ‘protect’ strategy is projected to be robustly cost-effective for 21st century SLR^3,4^, making it likely that other affluent coasts will follow suit^5^.

Transformational adaptations like ‘accommodating’ to SLR by (partly) retreating from high-risk areas or, conversely, ‘advancing’ the first line of flood defense seawards are gradually entering policy arenas^2,6^. In the Netherlands, a wealthy, urbanized delta with a strong tradition in flood defense systems, high SLR scenarios have sparked a heated debate: *Should we follow the historical path of building ever-higher dikes to protect current and continued investments behind these dikes, or should we opt for transformational alternatives?* Ultimately, many global deltas will face a similar question. Highly urbanized economies that are locked-in along the coast will be exposed to unprecedented growing flood hazard^7^. An example is the USA, where increasing flood risk leads to an alarming decrease in flood insurance coverage and affordability^8^. The Dutch debate is therefore informative to deltas worldwide.

The Dutch SLR debate (Supplementary Table 1) polarized around two extreme strategies: *Protect* vs *Accommodate-Retreat*. *Protect* aims to ‘hold-the-line’ through raising flood defenses and other technical solutions. Conversely, *Accommodate-Retreat* advocates nature-based solutions and ‘living-with-water’ instead of fighting it^9^. While both strategies aim to protect economic development, neither sufficiently considers how economies are bound to respond, for example via mechanisms like restructuring of economic activities, repricing, and reinvestment^10–12^. Cities are assumed to simply follow the adaptation strategy: either remain concentrated in their current locations (*Protect*), or shift to higher grounds (*Accommodate-Retreat*). However, history worked differently: flood protection followed economic development, in turn boosting the growth of population and capital in hazard-prone areas^12,13^. We argue that coastal adaptation debates should consider the economy as a dynamic rather than a static factor.

We advocate for extending the adaptation dialog with a perspective in which economic and financial mechanisms are thoroughly integrated, acknowledged, and understood. Finance and economics entail forces that will follow and steer; they can influence and be influenced by SLR and adaptation policies. In this paper, the term ‘economics’ refers to the real economic system (households, firms, governments) with its flows of production, consumption and distribution of all goods and services. ‘Finance’ refers to the financial system (financial markets, investments, banking, insurance) and its monetary flows. We not only argue that financial and economic mechanisms are important, but also explore what the important mechanisms are. This exploration is substantiated by literature and 14 semi-structured interviews (Supplementary Note 1) with economic and financial sector specialists, ranging from private (banking, insurance, investment) to public (financial stability, spatial planning) and academic research (Supplementary Table 2). This study provides an overview of the economic and financial mechanisms triggered by sea level rise adaptation strategies, supported by stakeholder insights. We conclude with a research agenda for urbanized coasts worldwide.

## Strategic directions for coping with sea level rise in The Netherlands

Exposure to severe floods has been the ‘norm’ for the Netherlands for centuries. To increase agricultural productivity, the Dutch started draining (and mining) peat swamps in the West of the country from 1000 AD onwards. Consequently, the peatlands subsided, requiring even more drainage and higher dikes. Later technological breakthroughs (windmills in the 15th century, steam pumps in the 19th century) enabled the reclamation of deep polders (to 6.8 m below present sea level). This satisfied the increasing demand for land, while the increasing economic activity justified the higher pumping and flood protection costs. In the 20th century, this culminated in the damming and reclamation of large portions of the Zuiderzee that threatened Amsterdam, followed by the closure of several estuaries in the Southwest^14^.

**Fig. 1:**
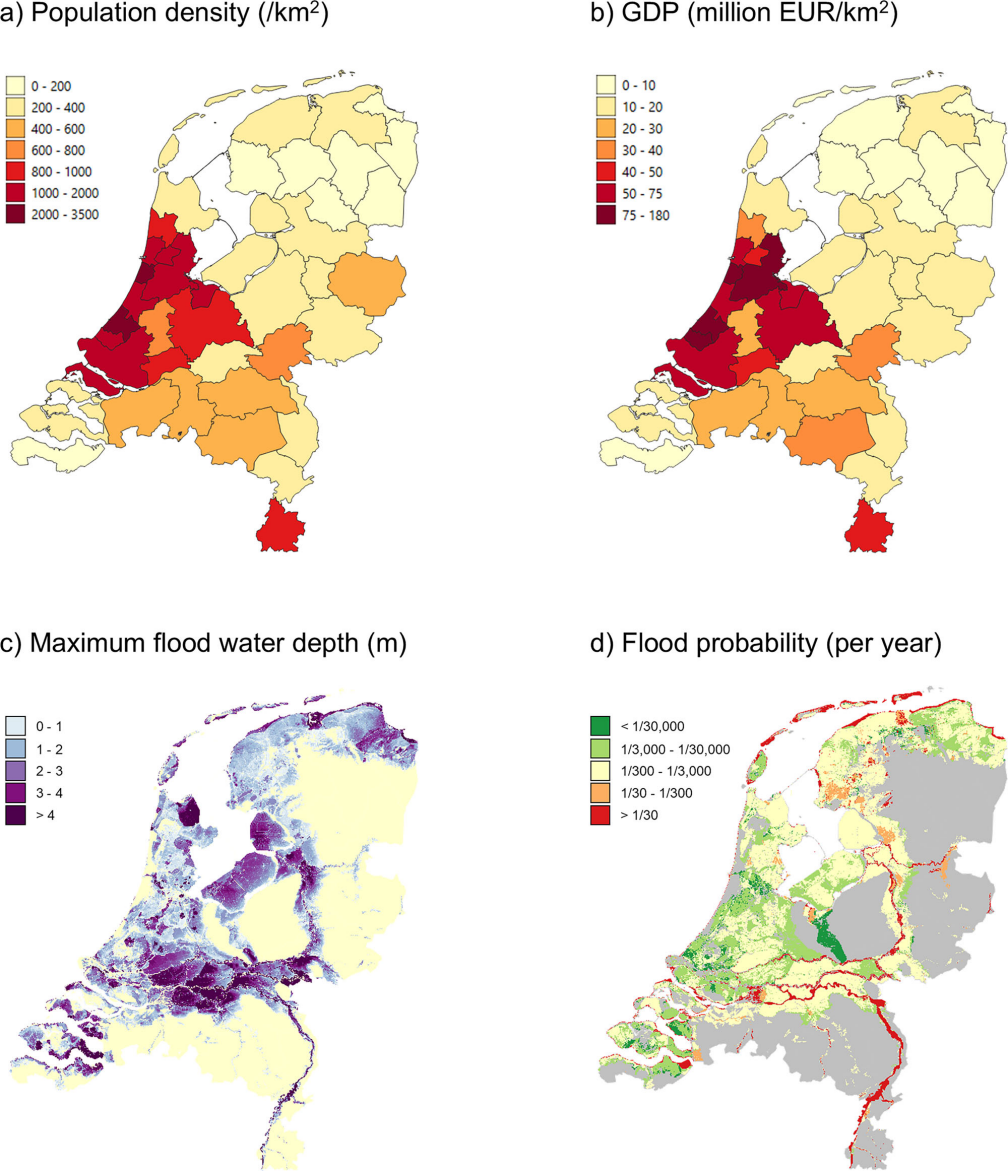
Socioeconomic and flood risk characteristics of The Netherlands. **a** Population density per land area (/km^2^) in 2024^18^, **b** economic output per land area (million euro/km^2^) in 2022^18^, **c** maximum flood water depth from river and coastal flooding (m) in the present climate (for the 1:100,000 year flood event) and **d** expected flood probability when flood protection standards are met in 2050. Source data (**c**, **d**): Ministry of Infrastructure and Water www.basisinformatie-overstromingen.nl.

At present, some 26% of the land area in the Netherlands is below sea level, and about half is exposed to river or coastal flooding (Fig. 1d). Almost half of the population and economic activity is concentrated in the low-lying, flood-prone, western part of the country: the *Randstad (‘Ring city’) area* (Fig. 1a, b). The high concentration of physical capital, companies, consumers, and workers in this area comes with considerable ‘agglomeration economies’^15,16^. Agglomeration economies refer to benefits that firms and citizens gain from being clustered together, including high-quality infrastructure and public services, a large pool of (multinational) firms and laborers, and attractive urban centers^17^. It is unsurprising that the flood safety standards (1000–30,000 year event) for levees and storm surge barriers protecting the Randstad agglomeration are amongst the highest in the world (Fig. 1d). National expenditures on flood protection are currently in the order of 2 billion EUR/year, constituting some 0.5% of the national government budget (390 billion EUR/year in 2023), and some 0.2% of GDP (1068 billion EUR/year in 2023^18^). Flood safety standards are determined by law and supported by cultural values of solidarity and sharing protection costs. Governance in the Netherlands features a (slow) decision-making process aimed at a consensus and the socioeconomic structure combines the market-oriented Anglo-American model with a strong welfare state and social policies^19^.

**Fig. 2:**
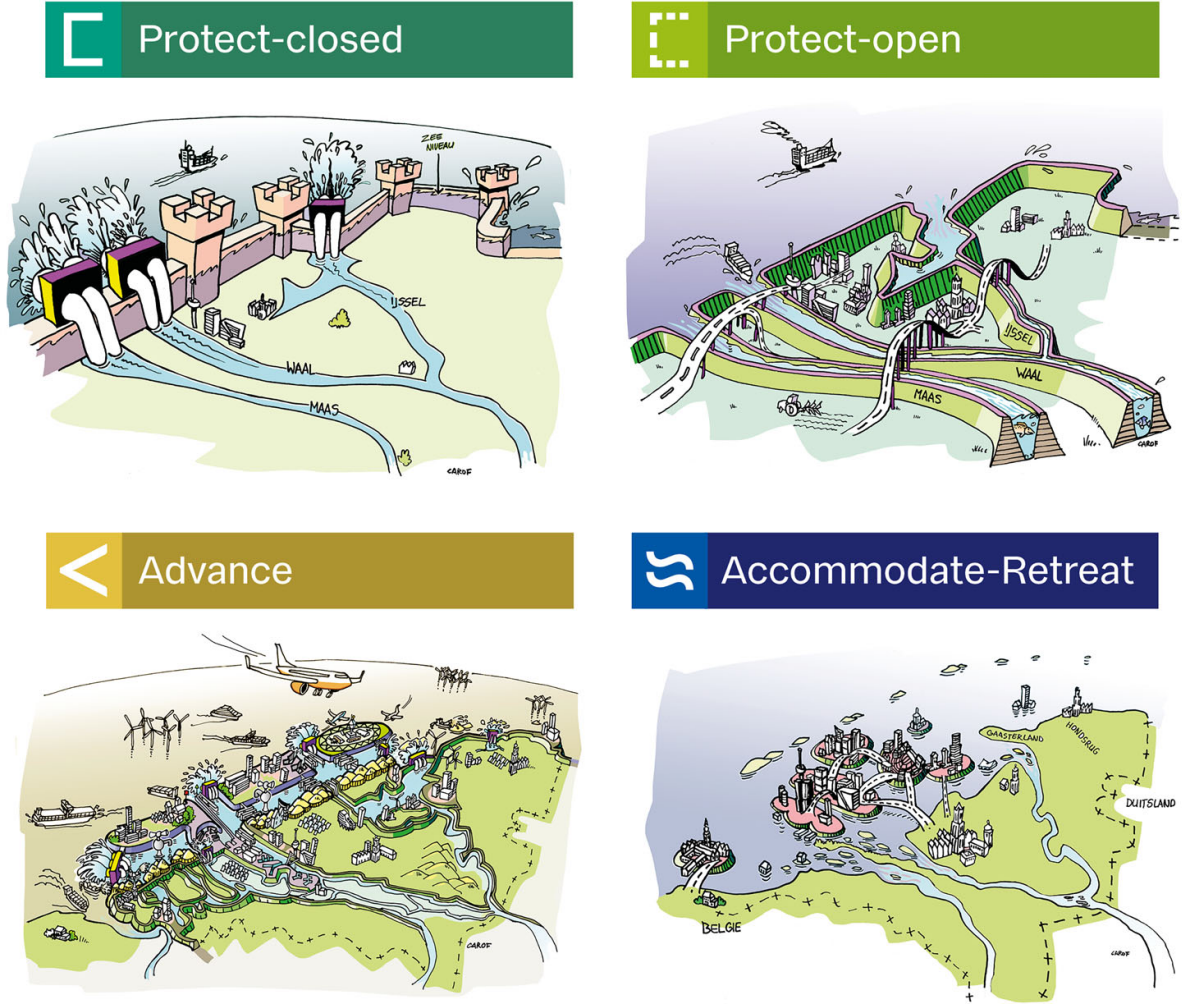
Four archetypical strategies for dealing with SLR in The Netherlands^22^. Image by Carof beeldleveranciers for Deltares^22^.

In 2018, the SLR debate intensified in The Netherlands, with a publication^20^ highlighting the possibility of more (>2 m instead of 1 m in 2100) and much faster SLR than was anticipated in the broadly accepted national adaptation strategy—the 2014 Delta Program^21^. Figure 2^22^ features four conceptual directions for SLR adaptation that arose during the subsequent exploration of possible solutions. This figure offers a visualization of the spectrum of possible strategies that could be taken in the future. The rest of this article discusses the two *Protect* strategies jointly and compares them with the *Advance* and *Accommodate-Retreat* strategies.

While there is more and more research on these strategies (Supplementary Table 1), the following questions remain: 1) what financial and economic *mechanisms* would each strategy trigger; 2) how would the autonomous forces fueling economic geographies facilitate or hinder these strategies^23^? Both questions ask about causal relations (‘mechanisms’) between adaptation and the economic and financial system. In question 1 the cause is the adaptation strategy and the effect is in the financial or economic domain, while in question 2 the cause lies in the financial or economic domain and the effect is on the adaptation strategy. Our answer to question 1 is summarized in Table 1 and further described below, and question 2 is addressed in section ‘different circumstances, different effects’.

**Table 1:** Financial and economic mechanisms and their expected individual, first-order effects triggered by the Accommodate-Retreat, Protect and Advance strategy, in the absence of actual, large-scale flood events

Mechanism	ACCOMMODATE-RETREAT STRATEGY			PROTECT STRATEGY	ADVANCE STRATEGY
	High area	Unprotected^a^, low area	Protected, low area	Protected, low area	Protected, low area, including new islands
Total National Flood Risk	Flood risk on a national scale decreases because of relocation to safer grounds; diversification of investments facilitates climate-resilient development			Risk may remain constant; but risk profile changes: decreasing probabilities (higher dikes) but increasing consequences (damage and casualties) in case of a dike breach	
Local Flood Risk	No flood risk	Short-run increase in risk for remaining assets/people; long-run risk reduction due to relocation	Risk may remain constant, but risk profile changes: decreasing probability (higher dikes) but increasing consequences (damage and casualties) in case of a dike breach		
*SOCIAL/DEMOGRAPHIC*					
Household welfare, including stranded home values	Increasing welfare for existing households	Decreasing welfare for existing households	Increasing welfare for existing households^b^	Short-run: unchanged welfare for existing households & decreased welfare for households in safe areas. Long-run: possible abrupt collapse	Increasing welfare for households
Migration	In-migration	Out-migration (most mobile people first)	In-migration^b^	Intensified in-migration to hazard-prone areas due to levee effect and the agglomeration benefits	In-migration due to levee effect and the agglomeration benefits
*LAND USE/INVESTMENTS*					
Investments	Increasing	Decreasing	Increasing^b^	Increasing and clustered in better protected but still hazard-prone areas	Increasing
Land prices	Increasing	Decreasing	Increasing^b^	Increasing, especially in current agglomerations like the protected Randstad area	Increasing, with a slight dampening effect of new islands (if developed along the Randstad area)
Land use	Less nature & agriculture, more residential, industrial & commercial	More nature and (extensive) agriculture, less residential, industrial, commercial Ultimately: mainly water and nature (full retreat)	Less nature & agriculture, more residential, industrial & commercial	Increased density of urbanization	Business as usual
Agglomeration economies	Decreasing urban/economic concentration of the existing Randstad and, thus, decreasing agglomeration economies. This may, in the long run, be (partially) compensated for by growing agglomerations on higher grounds outside Randstad			Continued urban/economic concentration in the low lying Randstad area, further strengthening agglomeration economies there.	
*FINANCIAL STABILITY*					
Portfolio risk exposure of investors and lenders	None	Low, decreases	Medium, increases	Short-run increases due to changing risk profile; long-run: possible abrupt collapse	
National financial stability	Short-term: instability concerns about transitions risk; long-term: decreasing concerns about the impacts of catastrophic flood events			Short-term: no concerns about transitions risks; long-term: increasing concerns about the impacts of catastrophic flood events	
*NATIONAL BALANCE SHEET*					
Expropriation costs	None	High	None	None	None
Economic output (GDP)	Strongly increases	Strongly decreases	Increases	Continuation of current growth path	Growth possibly beyond current growth path
Flood protection costs	None	Low	High and increasing	High and increasing	High and increasing

^a^Unprotected’ here refers to an absence of protection by dikes, in which case buildings might still be protected by building measures such as flood proofing or floating buildings.

^b^The mechanisms under ‘protect’ also apply here, possibly even in stronger form because of the reduced quantity of protected low-lying land. Interviewee 11 argued that keeping the low-lying Randstad while retreating/accommodating from the other low-lying areas would lead to more risk concentration than in the protect strategy, where all low-lying areas would be protected.

## Mechanisms triggered by the accommodate-retreat strategy

Under the *Accommodate-Retreat* strategy, large-scale flood defenses are no longer raised to adapt to SLR^24^. In addition to departing from the legacy of strengthening cost-effective, large-scale flood defense systems, this strategy deviates radically from two historical trends: 1) *increasing land area* through land reclamations; 2) *converting land to more productive uses* (less agriculture and nature, more residential and commercial)^25^. We assume the government effectively communicates which particular areas will no longer receive flood protection upgrades, and that damage from major flood events will not be compensated.

The economic and financial effects triggered by this strategy would be unevenly distributed in both space and time. In the short run, in exposed areas, real estate values would decline^26,27^. This could sort low-income households into these risky places or, if planning restrictions are put into place, steer people, jobs, and capital to higher grounds (Interview 2,5,7,11,13^28^) thereby raising real estate values there. Either way, the high agglomeration economies currently enjoyed in the low-lying Randstad area would decrease, and the national economy might suffer (Interview 5,11). In the long run, a declining economic output of the Randstad might be offset by increasing agglomeration economies in the destination areas^12^. Note, however, that new agglomerations take time to establish^28^. Overall, the nation-wide flood risk would decline. With proper stress testing, this should enhance trust, maintain the investment climate and contribute to financial stability.

Unlike the archetypical depiction of this strategy (Fig. 2), the abandonment of large-scale flood defenses is typically not envisioned for the most urbanized parts of the low-lying Randstad area^24^. This is because the government would most probably feel morally and politically obliged to (at least partly) compensate the ensuing short-term devaluation of real estate prices. Although marketable permits and development rights that could be swapped to less climate-sensitive locations could be cost-effective^29^, the short-term (transfer) cost of such an a bail out would be considered too high vis-à-vis the uncertain benefits in the distant future.

We therefore assume that *Accommodate-Retreat* only applies to the low-lying parts of the country, outside the Randstad^24^. This would create three areas: 1) Unprotected-Low; 2) Protected-Low (Randstad); 3) High (Table 1). Each of these areas would move towards a new economic equilibrium. With SLR, the Unprotected-Low areas would gradually become less safe and could be repurposed in stages^30^. Households and businesses would require relocation at an estimated flood risk of 1:200 years^24^. Intensive agriculture (at ~1:50 years, ibid.) and extensive agriculture (at 1:5 years, ibid.) would follow next. Over time, extensive tourism, nature, and eventually, only water would remain, offering new ecosystem services but contributing little to GDP.

Eventually, if the relocation is orderly, gradual, and timely, the long-term impact on the national economy might be positive because of the reduced flood risk to economic activities (Interview 4,9,11,14). The main difficulty lies in how to organize such a transition. The financial transition risks include the risk of stranded assets at old locations and the need for large capital investments in new locations (Interview 7). The transition may also have unequal wealth impacts: favoring home-owners and firms in the protected areas over those in the unprotected areas, and favoring mobile workers and firms (knowledge sector) over immobile workers and firms (Interview 11,12). This will come with substantial political risks (Interview 3,12).

## Mechanisms triggered by the Protect strategy

The *Protect* strategy preserves the current land area by continued or accelerated flood defense development. There are two *Protect* alternatives: 1) *Protect-Open*, keeping the river mouths open, requiring massive dike strengthening along the rivers (Fig. 2), and; 2) *Protect-Closed*, closing-off these river mouths, requiring massive pumping capacity (with high energy use) and space for water storage and causing severe ecosystem disruption (Fig. 2). Either alternative continues the historic trend of responding to increased flood risk via large scale, cost-effective technical protection measures, and therefore follows the same economic rationale. The additional (true resource) cost of protecting against 5.4 m SLR (in 2200) is estimated at 1.1-1.3 billion EUR/year^31^ in addition to the current flood risk expenditures of 2 billion EUR/year. Our experts considered this affordable (Interview 3,8,9,11,13).

What about the economic effects of this strategy? In the medium run, until 2100, the well-protected Randstad would likely keep attracting people, jobs, and capital to its low-lying cities, assuming no devastating flooding occurs. Increased capital at stake may justify higher than present protection levels due to the ‘safe development paradox’^32^. While net risks (expected annual damage) may remain stable, the risk *profile* would gradually progress towards lower probabilities and higher consequences, worsening a catastrophe, should defenses fail.

How international financial markets would ultimately respond to this changing risk profile is uncharted territory^33^. The Netherlands would likely pay an interest rate premium for such tail-risk catastrophic events (Interview 3,5,7)^34^. Thus far, flood risk has not become a barrier for investments in the Netherlands or impacted the national credit rating, which is currently triple-A. Our interviewees (5,6,7,10,14) judge the international sentiment as being positive relative to other countries; the financial markets trust in the ‘Dutch water solution’.

The keyword is indeed ‘trust’: trust in technical and institutional capacities and our ability to imagine how these capacities and risk profiles shift in a new climate reality. Experiencing a major flood event would undermine the trust in the current *Protect-Strategy* (Interview 5,7,9)^23,27^. At the same time, for the already well-protected Netherlands, it might suffice to be *relatively safe* compared to other coastal zones to attract international capital, but at the risk of locking itself into high sunk costs in the long-run^12^.

## Mechanisms triggered by an Advance strategy

The *Advance* strategy (Fig. 2) combines the construction of a second, higher, and stronger ‘defense line’ seaward, with the development of new barrier islands. The main technical advantage of this strategy is that the area between the old and new coastline can be used to buffer extreme river discharge^35^, so that the present land use in the country can be maintained. The buffer also keeps salinization in check, benefiting agriculture and nature. This mainly continues the historic trend of increasing land area. This trend can also be seen globally. In the last 30 years, heavily urbanized deltas, including Singapore, Dubai, and Rotterdam, have reclaimed more than 14,000 km^2^ of land from the sea^2^.

While many of the economic mechanisms for this strategy resemble *Protect*, some mechanisms are specific to *Advance*. We assume here that this strategy means that new land is created. On this land, new economic activities can be developed. The land could, for instance, be used for housing, industry, airports etc. Current real estate prices in the Randstad area are high and rising. The newly reclaimed land may relieve the tight property market and put downward pressure on these prices (Interview 2,13). Current agglomeration economies could be further strengthened. Furthermore, Hinkel et al.^5^ argue that, generally speaking, *Advance* may be easier to finance than *Protect* or *Accommodate-Retreat*, because of the revenue stream created by the economic activities deployed on the new land.

Major downsides of this strategy are the massive use of construction resources and the disruptive impact on the natural coastal ecosystems. The historical Zuiderzee and Delta Work projects radically transformed the ecosystems and continue to require efforts to find a new desirable eco-balance^36^.

## Different circumstances, different effects

So far, we addressed the financial and economic mechanisms that may be triggered by different SLR strategies in the Netherlands, from the perspective of current (positive) economic growth rates underpinned by existing technologies and value systems. Yet these conditions may change, radically affecting the adaptation strategies’ financial and economic effects and, hence, their uptake.

A first example of such a condition is economic growth. The higher this growth, the higher the rise in real estate prices. In *Accommodate-Retreat*, real estate in low-lying areas needs to be expropriated, and new real estate needs to be built in safer areas. The higher the real estate prices, the more expensive the buyouts become, which is a transfer cost of money that is paid from the government to owners of real estate. Depending on the government budgets and, ultimately, political priorities, some buyouts may become infeasible. Conversely, in *Advance*, real estate is developed instead of expropriated. The higher the real estate prices, the higher the revenues of new developments, and the better, ceteris paribus, the financial business case for this strategy, because it provides a funding mechanism to pay for the infrastructure.

A second condition that would affect how different adaptation strategies unfold is technology. An example is technology that reduces travel costs, like high-speed rails, autonomous vehicles, or virtual offices. The faster the technological progress and uptake of such innovations, the smaller the need for households and companies to physically cluster^17^ decreasing the positive spillovers and real estate price premiums of agglomerations^37,38^. This would diminish the ‘business-case’ of strategies protecting or expanding such agglomerations (i.e., *Protect* and *Advance*) and benefit (in a relative sense) strategies that reduce the agglomeration (i.e., *Accommodate-Retreat)*.

A third condition that might affect the financial and economic outcomes of different SLR strategies is society’s preference to build with nature. Whatever the origins (e.g., belief in its long-term effectiveness, distrust in technological solutions, health benefits of green environments, living in natural surroundings), the higher this preference, the higher the willingness to pay for nature-based solutions. This would improve the business case for *Accommodate-Retreat* and worsen, relatively speaking, that of *Protect* and, particularly, *Advance*.

## A research agenda for wealthy, urbanized coasts

We conclude with five insights that shape an economic research agenda for SLR adaptation in wealthy, urbanized coasts:

1. Coastal adaptation planning must explicitly consider the economic mechanisms to be triggered by each strategy. It should explore how climate adaptation dynamically affects prices, investment flows, production (and the other mechanisms of Table 1) over space and time.
2. The coastal adaptation strategy must be trusted by economic and financial markets. Coastal areas must therefore provide evidence that economic and financial impacts, also in catastrophic scenarios, will remain within acceptable levels^34^. For this purpose, new stress-testing methodologies need to be developed, because the pioneering work in this field^39–41^ is still limited in terms of covered sectors, transmission channels and forward-looking perspective^42^. Present model outcomes still seem inconsistent, e.g., the IMF^33,40^ macro-economic model finding a very large (14% of GDP) impact but their credit risk model finding a small banking impact (0.6 percentage point on core capital) for the same flood scenario in the Netherlands. Innovative analysis of climate risk transmission in financial networks is emerging^43^, but there are no attempts yet to extend it to the analysis of the adaptation strategies and their impacts on the financial system. Already, the IMF recommends the Netherlands to bring long-term flood scenarios into macroprudential oversight^33^, which, with SLR, becomes increasingly applicable to coastal countries globally.
3. A key characteristic of the Dutch debate is that the different strategies are not assessed with the same criteria, because advocates of each strategy use assessment criteria that align with different worldviews. For example, *Protect* aligns with trust in technological solutions, and emphasis on technical feasibility and economic efficiency up to 5 m of SLR. *Accommodate-Retreat* aligns with a preference for nature-based solutions and emphasis on how technological solutions have limits and can disrupt ecosystems^9^. While assigning weights to assessment criteria remains a subjective political choice, a first research step towards increased comparability could be the development of an indicator system that assesses all strategies on the same criteria and represents a broad welfare perspective on adaptation planning. This ‘broadness’ would need to apply to different domains (ecological, economic, social, etc.), space (regions, different people groups), and time (present and future generations)^44^.
4. Given the limited detail in the current strategies, the most feasible next step would be to apply multicriteria analysis (MCA) in combination with dynamic adaptive policy pathways (DAPP)^45^. To use Cost-Benefit Analysis (CBA) or a Real Option Analysis (ROA) that could be useful to aid decision making under deep uncertainty^46,47^, the strategies would first need to be elaborated into more concrete policy alternatives and monetary effects. This would also give the possibility to differentiate between strategies in space, a process that can be aided by land-use simulation models such as the LandUseScanner^11,48^. Some delicate points in CBA/ROA would be the choice of a discount rate that gives a consistent strategy on both the short and long term and how to duly incorporate the dynamic nature of the economy. Together, these techniques can give insight into the robustness of spatial and temporal strategies against the background of uncertainty of SLR and economic change.
5. Adding an economic and financial perspective helps to identify concrete actions that mix elements of opposing adaptation strategies. Consider the idea of better connecting the Randstad with higher ground and expanding the agglomeration benefits to safer parts of the country through investment in (high-speed) rail connections. The idea of ‘investing in higher grounds’ intuitively fits *Accommodate-Retreat* in which some low-lying areas would ultimately be repurposed or ‘given up’. However, this idea fits just as much in *Protect*, in which both the lower and higher areas are maintained, while spatially diversifying the economic activities (and therefore the financial risk) over flood-prone and safer grounds.

We hope that bringing in an economic and finance perspective will help advancing the SLR debate toward novel and broadly supported policy mixes.

## Supplementary information

Supplementary information

## Data Availability

The interview notes are not publicly available to protect the identity of the interviewees, but they are available from the corresponding author upon reasonable request and with the permission of the interviewee.
