# Supplementary material for: How an economic and financial perspective could guide transformational adaptation to sea level rise
Source: NPJ Clim Action. 2025 Sep 24;4(1):89. doi: 10.1038/s44168-025-00297-4 (PMC12460159; doi:10.1038/s44168-025-00297-4)
Supplement: Supplementary file 1 — Supplementary information [file 44168_2025_297_MOESM1_ESM.docx]

**Supplementary information to: how an economic and financial perspective could guide transformational adaptation to sea level rise**

Kees C. H. van Ginkel^1,2,*^, Bart Rijken^1^, Marco Hoogvliet^1^, Wesley van Veggel^1^, W. J. Wouter Botzen^2^, Tatiana Filatova^3^

1. Deltares, Delft, The Netherlands
2. VU University, Amsterdam, The Netherlands
3. TU Delft, Delft, The Netherlands

***Corresponding author**
Kees C.H. van Ginkel, [kees.vanginkel@deltares.nl](mailto:kees.vanginkel@deltares.nl)
Deltares, Department of climate adaptation and disaster management, Boussinesqweg 1, 2629 HV Delft, The Netherlands.

Supplementary Table 1 Financial and economic considerations in Dutch publications on strategies for dealing with high-end SLR

| **Ref.** | **Main outcome** | **Economic aspects** |
| --- | --- | --- |
| 1 | Adaptation tipping points indicating when thresholds in the water system are reached | None |
| 2 | 4 strategic directions one could take (Figure 2 of the main article) | Costs of the strategies are very briefly discussed, but not specified or quantified |
| 3 | Building blocks that would fit under each strategic direction | Does mention that finance and economics play a role, but not how. |
| 4 | Synthesis report of the three reports below (5-7) | See below |
| 5 | Protect - This solution is technically and financially feasible | Implementation costs of four protect alternatives for 2100 and 2200 (163-217 billion EUR), each including costs of dikes, storm surge barriers and pumps.  Number of houses that would need to be relocated. |
| 6 | Advance - Plan for construction of a new coastal defense and water retention area in South-Western part of The Netherlands | Construction costs: 30-35 billion EUR, maintenance costs: 250-350 million EUR/year.  New residential/economic developments are not part of this plan. |
| 7 | Accommodate - Giving up the Randstad is not a realistic option considering the agglomeration benefits | Essay on the agglomeration effect, and how a transition to higher grounds could look like (Van Haaren et al., 2023) |

Supplementary Table 2 Interviews with researchers and financial sector representatives

|  | **Date (Y-M-D)** | **Organization type** | **Interviewees core expertise** |
| --- | --- | --- | --- |
| 1 | 2024-04-02 | Private - insurance | Climate finance, environmental economics |
| 2 | 2024-04-25 | Research - spatial planning | Land, real estate and urban development |
| 3 | 2024-04-26 | Public - regional economics | Climate impacts, economic analysis, cost benefit analysis |
| 4 | 2024-05-08 | Research - regional economics | Regional economic dynamics |
| 5 | 2024-05-16 | Private – bank | Micro-economic impacts (households, businesses) of climate risk |
| 6 | 2024-05-23 | Private – pension fund | Climate change impacts on investment decisions, portfolio exposure |
| 7 | 2024-05-23 | Public – financial sector | Financial stability assessment, supervision |
| 8 | 2024-05-27 | Private – economic analysis | Flood risk management, cost-benefit analysis |
| 9 | 2024-05-27 | Research – environmental economics | Flood risk management, cost-benefit analysis |
| 10 | 2024-05-29 | Research – economics | Real estate markets |
| 11 | 2024-06-03 | Research – urban economics | Climate risk, real estate markets |
| 12 | 2024-06-10 | Research – international economics | International and monetary economics, regional and spatial economics |
| 13 | 2024-06-28 | Public – spatial planning | Climate and built-environment, flood safety |
| 14 | 2024-09-26 | Private – bank | Macro-economics and (public sector) finance |

**Supplementary Note 1: interview questions**

1. Could you tell us something about your expertise, and about the organization you are affiliated with?
   1. Would you characterize your organisation as public sector, private sector, or a research institute?
   2. What is your current role/position within your organization?
   3. How would characterize your knowledge field or expertise?

*The interviewer shows a picture with the KNMI scenarios for sea level rise in the Netherlands, including the scenarios with much and fast SLR due to rapid disintegration of the Greenland and West-Antarctic icesheets (Supplementary Figure 1).*

1. *The interviewer makes notes about the response to this, and if needed asks the question*: did you hear about this before? To what extent should we plan for this type of tail-risks? And do you think it is rational to prepare for this?
2. Say these scenarios would become reality, what would be the impact to the Dutch macro-economy (or more specifically: your sector)?

*The interviewer introduces the four strategic adaptation directions using figure 2 (main article) and makes notes about the response to this. Upon request, the interviewer further clarifies the strategic directions, using the latest synthesis reports for protect-advance-accommodate (Supplementary Table 1 – report 5-7)^[[1]](#footnote-1)^.*

1. Did you hear about these four strategic directions before, and if so: what do you think of it?
2. What are the key transmission channels from adaptation strategies (e.g. protect, accommodate) to economic development, and vice versa?
   *The interviewer introduces an example and makes sure that at least the protect and accommodate strategies are discussed.*
   1. *Solution -> economy –* How would implementing each strategy effect society and/or the economy? Please describe each mechanism separately.
   2. *Economy -> solution space –* What economic/financial development could contribute to, or be a barrier to each strategy? Please describe each mechanism separately.
3. *If the interviewee is very sceptical about one or more solutions, the interviewer asks:* How would the world need to look to make this a realistic strategic direction? What are the socio-economic conditions that would enable a certain direction? Are there other places in the world where this strategy is more likely?
4. Did we miss anything important in this questionnaire? What question would you ask yourself if you were me?


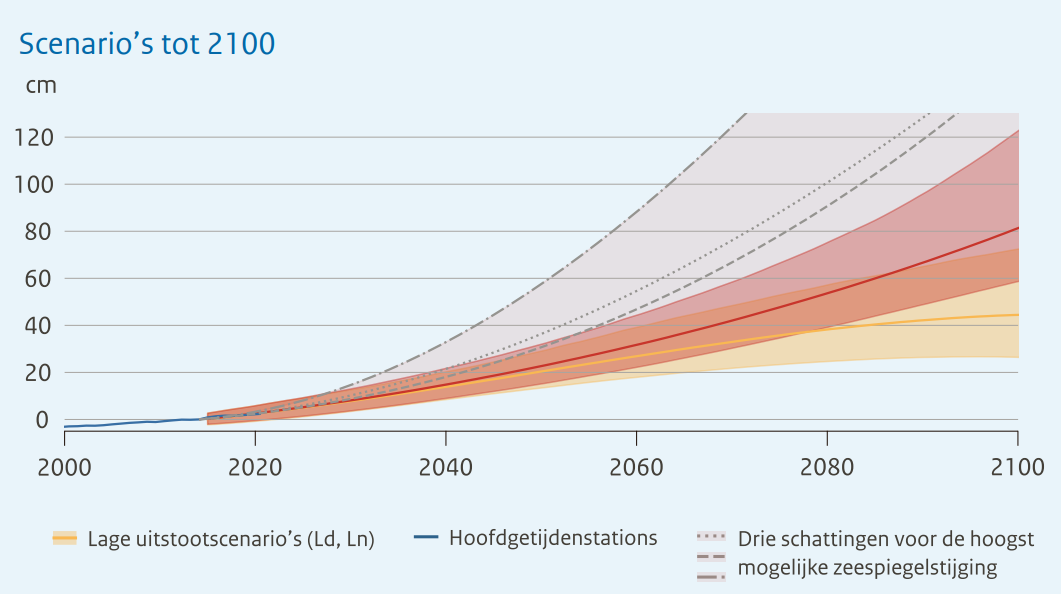


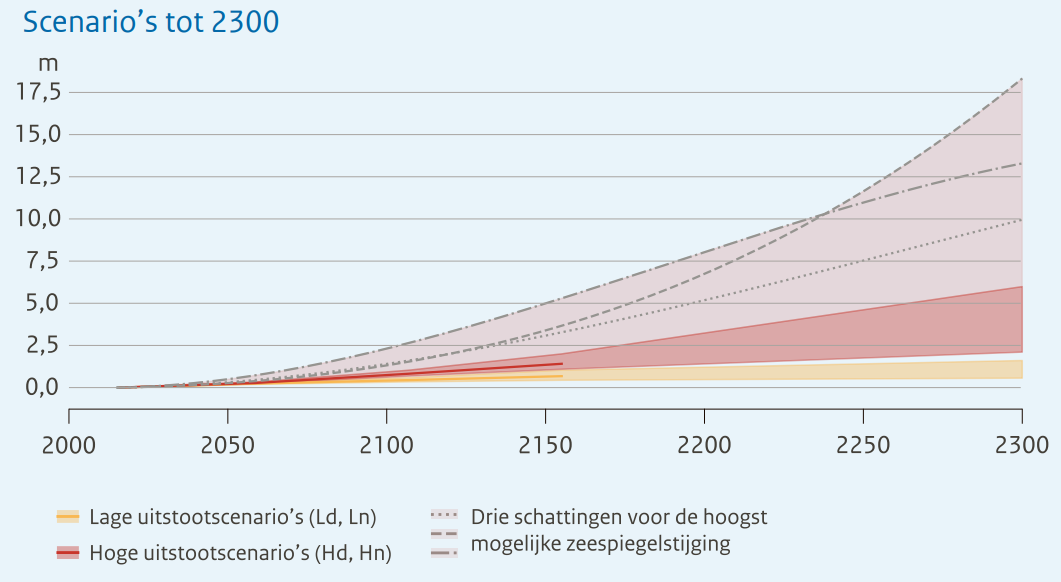


Supplementary Figure 1 The scenarios shown to the interviewees after question 1. These show projected sea level rise along the Dutch coast for a low (Ld) and high (Hd) emission scenario, both the median and 90% confidence interval, as well as three high-end estimates for ‘the highest possible sea level rise’ due to icesheet instabilities. Source data: ^8^, source graphic: ^9^

**Supplementary References**

1. Haasnoot, M., Bouwer, L., Diermanse, F., Kwadijk, J., van der Spek, A., Oude Essink, G., Delsman, J., Weiler, O., Mens, M., ter Maat, J., Huismans, Y., Sloff, K. & Mosselman, E. *Mogelijke gevolgen van versnelde zeespiegelstijging voor het Deltaprogramma. Een verkenning.* Report No. 11202230-005-0002 (Deltares, 2018).
2. Haasnoot, M., Diermanse, F., Kwadijk, J., de Winter, R. & Winter, G*. Strategieën voor adaptatie aan hoge en versnelde zeespiegelstijging. Een verkenning.* Report No. 11203724-004 (Deltares, 2019).
3. Haasnoot, M. & Diermanse, F. (ed.). *Analyse van bouwstenen en adaptatiepaden voor aanpassen aan zeespiegelstijging in Nederland.* Report No. 11208062-005-BGS-0001 (Deltares, 2022).
4. Deltaprogramma, Ministerie van Infrastructuur en Waterstaat. *Ruimte voor zeespiegelstijging. Een verkenning van denkrichtingen om Nederland ook op lange termijn veilig en leefbaar te houden bij zeespiegelstijging* (Ministerie van Infrastructuur en Waterstaat, 2024).
5. Witteveen & Bos. *Technisch-fysische uitwerking Oplossingsrichting beschermen*. Report No. 135942/23-017.432 (Witteveen & Bos, 2024).
6. Hekman, A., Booister, N., Vrinds, T., Kruitwagen, G., Steijn, R., Rijks, D., Huisman, B., Luijenduik, A., Diermanse, F., van Spengen, J., van Broekhoven, P., van der Werf, M., Koreman, K., Harman, J., Lodder, Q., Roos, I. & van Alphen, J. *Technisch-fysische uitwerking oplossingsrichting Zeewaarts*. Report No. 51013204 (Sweco, 2024).
7. Zanting, H. A. & Bouw, M. *Oplossingsrichting ‘meebewegen’. Verkennend onderzoek voor het kennisprogramma zeespiegelstijging*. Report No. AKDRCXACJ4FW-990297605-866:1.0 (Arcadis, 2023).
8. van Dorland, R., Beersma, J., Bessembinder, J., Bloemendaal, N., van den Brink, H., Brotons Blanes, M., Drijfhout, S., Haarsma, R., Keizer, I., Krikken, F., Le Bars, D., Lenderink, G., van Meijgaard, E., Meirink, J. F., Reerink, T., Selten, F., Severijns, C., Siegmund, P., Sterl, A., Overbeek, B., de Vries, H., Wichers Schreur, B. & van der Wiel, K. *KNMI National Climate Scenarios 2023 for The Netherlands* (KNMI, De Bilt, 2023).
9. KNMI. KNMI’23-klimaatscenario’s voor Nederland. KNMI-Publicatie 23-03 (KNMI, De Bilt, 2023).

1. Regarding accommodate-retreat, the interviewer introduces the outcome of report 7 (Supplementary Table 1), that giving up the Randstad area is not seen as a realistic option, creating a difference between ‘high area’, ‘unprotected, low area’ and ‘protected, low area’, as in Table 1 of the main article. [↑](#footnote-ref-1)
